# Spasmolytic Effect of *Grewia asiatica* Fruit Extract on Isolated Smooth Muscles is Mediated via Multiple Pathways

**DOI:** 10.1155/2021/5583372

**Published:** 2021-09-13

**Authors:** Muhammad Nabeel Ghayur, Saeed Ahmad, Anwarul Hassan Gilani

**Affiliations:** ^1^Department of Biological and Biomedical Sciences, Aga Khan University, Karachi 74800, Sind, Pakistan; ^2^Kentucky College of Osteopathic Medicine, University of Pikeville, Pikeville 41501, KY, USA; ^3^Department of Public Health and Nutrition, The University of Haripur, Haripur 22620, Khyber Pakhtunkhwa, Pakistan

## Abstract

**Background:**

*Grewia asiatica* Linn, or phalsa, is a commonly consumed fruit in Pakistan. The fruit is employed in the traditional medicine practice of Pakistan as a smooth muscle relaxant in different gastrointestinal (GI) and cardiovascular diseases. In this investigation, we show the antispasmodic and vasorelaxant actions of *Grewia asiatica* fruit extract.

**Methods:**

A 70% methanolic crude extract of the plant material was prepared (Ga.Cr). Different isolated GI tissue preparations and endothelium-intact aortas from rats were utilized to observe the pharmacological actions of the extract.

**Results:**

Ga.Cr, in increasing concentrations, inhibited the spontaneously contracting rabbit jejunum. In an effort to determine the mechanism of this relaxant action, contractions were induced in jejunum and ileum tissues with K^+^ (80 mM). Ga.Cr was able to only partially inhibit these induced contractions indicating that the mechanism might not be completely through a blockade of Ca^2+^ channels (CCB). When tested on low K^+^-(25 mM) sustained contractions, Ga.Cr cumulatively suppressed these contractions (0.1–10 mg/ml), indicating an opening of K^+^ channels (KCO) as the mechanism. Cromakalim, a standard KCO, was also more specific in blocking low K^+^-induced contractions. For the effect in aorta tissues, Ga.Cr suppressed the agonist-induced contractions from 0.3 mg/ml to 10 mg/ml. Upon challenge with L-NAME, a nitric oxide (NO) blocker, the extract response curve shifted right, indicating vasodilation was mediated via endothelial NO.

**Conclusion:**

This study shows that GI antispasmodic and vasodilator activities of Ga.Cr may be mediated via a KCO mechanism in the GI tract and through the release of NO from vascular endothelium.

## 1. Introduction

*Grewia asiatica* Linn (family Tiliaceae) is a plant commonly known and cultivated in South Asia. It is known by the common name of phalsa in Hindi and Urdu. The plant grows into a small tree or shrub around five meters in height. The fruit from the tree, also known as phalsa (purple to black and 1–1.5 cm in diameter), is an edible berry that is very popular with the people of South Asia. It is known specifically as a cooling agent in the summer months, and people typically eat the berry raw or consume as juice or syrup. Not only is the phalsa fruit liked as an edible berry, but the berry and other parts of the plant, including the bark, leaves, buds, and roots, are used by traditional healers of the region for their medicinal benefit [1]. The bark and roots are known to relieve inflammation of joints and muscles, the leaves are useful for infectious skin conditions, and the fruit is known to be beneficial in indigestion, diarrhea, and cardiac/heart disorders [1–3].

Some of the pharmacological studies performed on the plant show its medicinal benefits in anxiety, depression, memory impairment [4], diabetes, hyperlipidemia [5–8], pain, fever, inflammation [7, 9, 10], and infections [11, 12]. In terms of the phytochemical constituents, prior studies report that *Grewia asiatica* has several polyphenols [13, 14], carbohydrates, tannins, vitamin C [15], and different amino acids [3].

We undertook this project to justify the use of *Grewia asiatica* fruit in gastrointestinal (GI) and cardiac disorders. In the course of our experiments, we found that the 70% aqueous methanolic extract of *Grewia asiatica* fruit exhibited antispasmodic activity in isolated GI tissue preparations and the vasodilator effect in isolated vascular tissues.

## 2. Materials and Methods

### 2.1. Animals

A determined effort was undertaken to minimize the suffering of the animals at every step of this project. Experiments were performed as detailed in the European Community guidelines, EEC Directive 86/609/EEC. Smooth muscle tissues were acquired from local rabbits weighing around 1 kg and guinea pigs with an average weight of .5 kg of both sexes were used. Sprague-Dawley rats of either sex, weighing around 200 g, were also used. These animals were kept in Aga Khan University's animal house where the temperature was kept between 23°C and 25°C, while the conditions were ensured to be pathogen-free. The animals were given water as needed. A standard diet was provided to the animals [16], though it was withdrawn a day before the experiment.

### 2.2. Drugs and Reagents

For the experimentation part, the following standard pharmacological agonists and antagonists were used: acetylcholine chloride (ACh), L-NG-nitro arginine methyl ester (L-NAME), phenylephrine (PE), potassium chloride, and verapamil (all from Sigma, USA). Tocris and RBI Chemicals were contacted for cromakalim and glibenclamide, respectively. For the physiologic salt solutions, Tyrode's and Krebs's solutions were used, the ingredients of which were acquired from Sigma Chemical Co. and Merck, Germany.

### 2.3. Crude Extract Preparation

Around 1 kg of phalsa berries were collected in Karachi, Sind, Pakistan (geographical coordinates: latitude 24°53′40.3224″N, longitude 67°3′48.906″E (degrees, minutes and seconds, DMS); or Lat 24.894534, Long 67.063585 (decimal degrees, DD)). A specimen of this was identified and sampled with the Herbarium of Department of Biological and Biomedical Sciences, Aga Khan University.

The phalsa fruit was cleaned, washed, and then lightly opened up with a pestle. For the process of making an extract, these were then dipped in a couple of liters of a mixture of 30% distilled water and 70% methanol (analytical grade from Merck Co., Darmstadt, Germany) for 72 hours. At the end of this period, the contents were filtered through a cloth filter, and the plant material was again dipped in a new supply of solvent mixture, twice, for 72 hours each. Finally, all the filtrates were collected and passed through Whatman filter paper. For the process of concentration, a rotary evaporator was used. This resulted in obtaining a viscous extract coded as Ga.Cr.

### 2.4. Rabbit Jejunum

We have performed this method of utilizing isolated rabbit jejunum tissues for pharmacological testing before [16]. To briefly describe the method here, pieces of intestinal jejunum around 2 cm long were acquired from rabbits and hung using cotton thread in glass muscle baths with Tyrode's at 37°C. Tyrode's solution was bubbled with carbogen gas. A preload of a gram was used on jejunum segments and were left to stabilize for half an hour and connected to oscillographs and isotonic force transducers (Harvard Apparatus, USA). The tissues were stabilized with a standard contractile agent ACh (1 *μ*M). Rabbit jejunum is a tissue that when set up like this elicits spontaneous rhythmic contractions that help in testing for both spasmolytic and spasmogenic agents. The muscle inhibitory effect of the extract was calculated in comparison to the baseline muscle contractility, while any possible spasmogenic effect was compared to the ACh maximum effect.

A potential spasmolytic response from the test extract was further investigated on contractions sustained in rabbit jejunum with the help of high K^+^ (80 mM) and low K^+^ (25 mM). Suppression of high K^+^ indicated a calcium channel blocking (CCB) effect, and suppression of low K^+^ indicated a relaxant effect mediated via the opening of K^+^ channels [17, 18]. The suppression of these induced contractions by Ga.Cr was calculated as compared to the effect exhibited by high and low K^+^.

### 2.5. Guinea Pig Ileum

We have described working with the guinea pig ileum in detail before [19]. In summary, 1-2 cm long pieces of ileum were obtained from guinea pigs. These pieces were maintained in tissue baths, and recordings were made as described above for rabbit jejunum. Tissues were kept for 30 minutes to normalize and then stabilized with repeated doses of a standard agent, ACh (1 *μ*M). A three-minute gap was allowed between administered concentrations of standard or test agents. Unlike rabbit jejunum, guinea pig ileum does not exhibit rhythmic contractions but only shows a flat baseline ideal for testing of potential spasmogenic agents. Spasmolytic activity can be tested with the help of an agonist. This was done the same way as described with jejunum tissues. Contractions were obtained with a high strength of K^+^. Later, the test substance was administered cumulatively to record for any relaxant activity.

### 2.6. Endothelium-Intact Rat Aorta

A previously described method was followed [20] with some changes [21]. In brief, aorta tissue was carefully obtained, so that the endothelial part stayed intact. Tissue of about 3 mm width was maintained at 37°C in Krebs's and aerated with carbogen gas. Baseline tension of a gram was used, and rings were left to normalize for half an hour. Any changes with the vascular muscle were recorded with the help of isometric force transducers (World Precision Instruments Fort 100), linked with Transbridge 4 M and captured through the CVMS data acquisition system. Standard vasoconstrictor PE (1 *μ*M) was used for tissue stabilization. While in poststabilization, the contraction was induced with the same agent. Acetylcholine (0.1 *μ*M) was utilized to test endothelial-mediated suppression of PE contractility. Later, the test extract was administered on PE contractions in increased concentrations. For any potential characterization of a vasodilator effect, L-NAME (0.1 mM; 60-minute pretreatment period) was used as an antagonist to block a potential nitric-oxide- (NO-) mediated vasodilator effect in the endothelium-intact preparations.

### 2.7. Representation of Results

The results are expressed as the mean ± standard error of the mean, or SEM, while “*n*” is the number of observations. The median effective concentrations (EC_50_) have 95% confidence intervals (CI). Curves were compared utilizing two-way analysis of variance (ANOVA) with *p* < 0.05 suggesting meaningful difference (GraphPad, USA).

## 3. Results

### 3.1. Rabbit Jejunum

**Figure 1:**
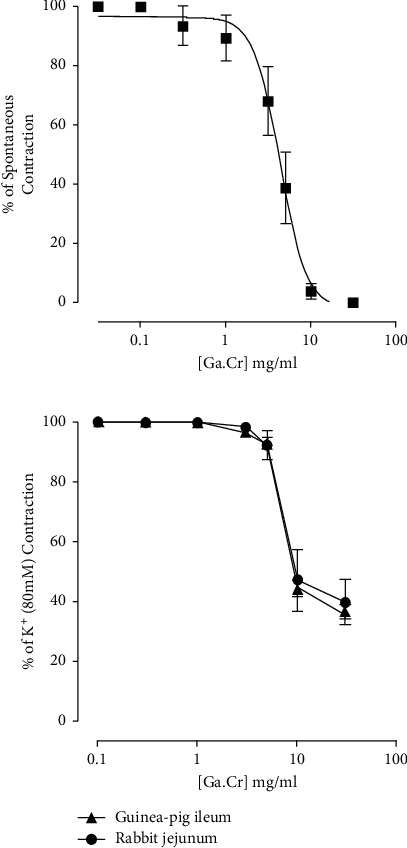
The graphs presenting the activity of *Grewia asiatica* 70% methanolic extract (Ga.Cr) on isolated intestinal smooth muscle preparations. The graph on the top shows the effect of Ga.Cr upon baseline rabbit jejunum spasmogenicity. The relaxant activity depicted is the percent of spontaneous contractions of the tissue (results are mean ± SEM for 7 observations). The graph at the bottom shows activity of the extract on high K^+^ (80 mM) spasmogenicity in jejunal and ileal preparations. The spasmolytic behaviour of Ga.Cr is % of agonist-sustained spasmogenicity (results are mean ± SEM for 3 observations).

**Figure 2:**
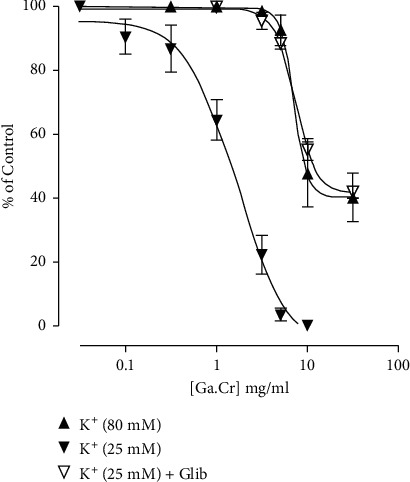
The graph showing how *Grewia asiatica* 70% methanolic extract (Ga.Cr) suppresses the spasmogenicity of K^+^ 80 mM and 25 mM (with and without the standard antagonist glibenclamide or glib 3 *μ*M) on jejunal tissues of a rabbit. Spasmolytic action of Ga.Cr is the percent of K^+^-induced contraction (results are mean ± SEM for 3 observations).

Ga.Cr exhibited a spasmolytic effect on the default spontaneously beating jejunal tissue when used in increasing concentrations from 0.3 mg/ml to 10 mg/ml. The EC_50_ for this effect was 4.4 mg/ml (3.5–5.3, *n* = 7). The extract concentration of 10 mg/ml completely suppressed the jejunum contractility (Figure 1). High K^+^ (80 mM) was administered to figure out the reason for this relaxation, and sustained contractions were obtained. Ga.Cr was then administered cumulatively on these contractions. The extract from 3 mg/ml to 30 mg/ml only partially suppressed K^+^ 80 mM contractions (Figures 1 and 2). The EC_50_ for this effect was 7.1 mg/ml (5.3–9.3, *n* = 3). In comparison, Ga.Cr (0.1–10 mg/ml) fully suppressed K^+^ 25 mM induced contractions with EC_50_ of 1.6 mg/ml (1.1–2.4, *n* = 3, Figure 2). The action of Ga.Cr on 80 mM compared to 25 mM was significantly different (*p* < 0.0001). The depressant action of the extract on 25 mM was blocked (curve shifted to the right; *p* < 0.0001 against the control curve) when challenged with glibenclamide (3 *μ*M) and was only mediated from 3 mg/ml to 30 mg/ml. The EC_50_ for this response was 7.3 mg/ml (6.4–8.3, *n* = 3, Figure 2).

**Figure 3:**
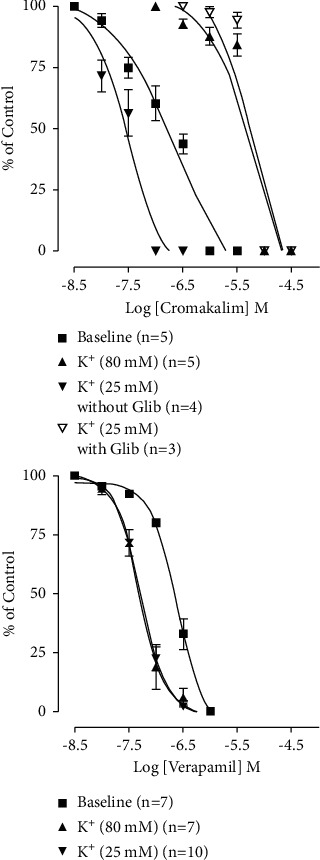
The graphs representing the activity of different standard spasmolytic agents on isolated rabbit jejunum tissue preparations. The graph on the top shows the effect of increasing concentrations of cromakalim upon resting baseline, K^+^ (80 mM) and K^+^ (25 mM) spasmogenicity, and K^+^ (25 mM) with and without the standard antagonist glibenclamide 3 *μ*M (glib) (results are mean ± SEM for 3–5 observations). The bottom graph showing the effect of verapamil on the resting spontaneous baseline, K^+^ (80 mM), and K^+^ (25 mM) spasmogenicity (results are mean ± SEM for 7–10 observations).

Some standard drugs, such as cromakalim and verapamil, were also tested for comparison with the extract. Cromakalim, 0.01–1 *μ*M, suppressed the rhythmic baseline contractions of the jejunum (Figure 3) with EC_50_ of 0.23 *μ*M (0.11–0.47, *n* = 5). Cromakalim was also cumulatively administered on K^+^ 80 mM. It inhibited these contractions from 0.1 *μ*M to 10 *μ*M. The EC_50_ was 7.4 *μ*M (3.6–15.0, *n* = 5). In comparison, when cromakalim was administered upon K^+^ 25 mM contractions (Figure 3), the relaxant effect came from 0.01 *μ*M to 0.1 *μ*M. The EC_50_ for this was 0.03 *μ*M (0.02–0.05, *n* = 4, *p* < 0.0001 when matched with the K^+^ 80 mM effect), and this was shifted to the right/blocked (effect from 0.3 *μ*M to 10 *μ*M, EC_50_ of 7.3 *μ*M (2.1–25.6, *n* = 3) when challenged with glibenclamide (3 *μ*M, *p* < 0.0001 compared to the control effect).

Verapamil showed its relaxant effect on jejunum baseline contractions from 0.01 *μ*M 1 *μ*M. The EC_50_ for this effect was 0.25 *μ*M (0.20–0.30, *n* = 7, Figure 3). Verapamil's effect on high and low K^+^-induced contractions (Figure 3) was exhibited at a similar range of concentrations (*p* > 0.05), from 0.01–0.3 *μ*M. The EC_50_ value for effect on K^+^ 80 mM was 0.05 *μ*M (0.04–0.06, *n* = 7), and the effect on K^+^ 25 mM was the same at 0.05 *μ*M (0.04–0.06, *n* = 10).

### 3.2. Guinea Pig Ileum

Ga.Cr did not show any stimulant effect on the ileal quiescent tone up to a concentration of 10 mg/ml (*n* = 3, data not shown). On the contrary, Ga.Cr exhibited a mild and partial relaxant effect (3–30 mg/ml, *n* = 3) on K^+^- (80 mM) sustained spasmogenicity (Figure 1). This effect had the same potency and efficacy shown by Ga.Cr in rabbit jejunum (*p* > 0.05). Because of this, further testing was done only in rabbit jejunum rather than both tissue preparations.

### 3.3. Endothelium-Intact Rat Aorta

**Figure 4:**
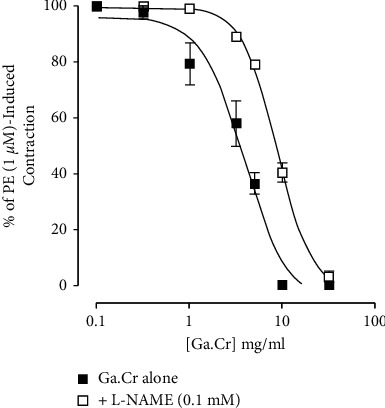
The graph showing the vasodilator effect of *Grewia asiatica* 70% methanolic extract (Ga.Cr) on isolated endothelium-intact rat aorta tissue preparation, with and without L-NG-nitro arginine methyl ester 0.1 mM (L-NAME). The vasorelaxant effect was quantified upon phenylephrine 1 *μ*M (PE) spasmogenicity. The vasorelaxant action of the extract was determined as a percent of PE-induced contractions (results are mean ± SEM for 4 observations).

Ga.Cr did not show any stimulant effect (*n* = 3, data not shown) on the default quiescent tone of the aorta. Later, contractions were induced by PE (1 *μ*M) in the aorta, which the extract, in increasing concentrations of 0.3–10 mg/ml, relaxed. The EC_50_ for this relaxant effect was 3.9 mg/ml (3.0–4.9, *n* = 4, Figure 4). This vasorelaxant action of the extract was repeated in tissues pretreated with the blocker L-NAME (Figure 4). This impacted the extract's vasodilator action as seen by the switching of the concentration-response effect (*p* < 0.0001 from control curve). The vasodilator action of extract in the presence of the blocker was mediated from 3 mg/ml to 30 mg/ml, while the EC_50_ for this was 8.7 mg/ml (8.0–9.4, *n* = 4, Figure 4).

## 4. Discussion

Phalsa berries are traditionally known to be beneficial for GI and cardiovascular ailments [1–3]. For this reason, a study was planned to investigate if there is any scientific basis for their use in GI disorders. Our results showed that a 70% aqueous methanolic crude extract of phalsa fruit concentration-dependently relaxed the spontaneous contractions of isolated rabbit jejunum tissues (Figure 1). The rabbit jejunum contracts in a spontaneous manner when stabilized and immersed in a physiologic salt solution under a controlled environment. This makes it useful in testing for potential GI spasmolytic agents [16, 22]. The crude extract was also tested on ileal preparations. Guinea pig ileum behaves very differently from the rabbit jejunum in the sense that it does not exhibit spontaneous contractions. This means that the guinea pig ileum is not the ideal tissue to test initially for spasmolytic agents (unless a standard agonist is used) but rather a good tool to test for the presence of spasmogenic characteristics [22]. Ga.Cr extract did not show any spasmogenic activity, further strengthening the result acquired in rabbit jejunum.

To characterize this spasmolytic activity of Ga.Cr extract, spasmogenicity was induced by K^+^ 80 mM in jejunum and ileum tissues. The extract was able to concentration-dependently relax; to a certain extent, these induced contractions in the jejunum and ileum preparations (Figure 1). This effect was seen only at higher concentrations in both the tissues (3–30 mg/ml), and the extract was unable to completely abolish the induced contractions. The extract reduced K^+^ 80 mM contraction in the jejunum to 40 ± 7.6% and in the ileum to 36.7 ± 2.4% of K^+^ maximum contraction. It was clear from this finding that the extract was unable to completely suppress K^+^ contractions. As the extract was equipotent in its response in the jejunum and ileum, only the jejunum tissue was selected for further testing.

To further research this spasmolytic action, Ga.Cr was administered on K^+^ (25 mM) spasmogenicity in rabbit jejunum. A suppression of K^+^ (80 mM) contractions normally indicates a Ca^2+^ channel blocking effect (CCB), while specific suppression of K^+^ (25 mM) contractions indicates a K^+^ channel opening (KCO) effect [18, 23]. The extract concentration-dependently completely relaxed these 25 mM contractions indicating that it can fully suppress K^+^ (25 mM) but not K^+^- (80 mM) induced contractions (Figure 2). This pointed towards a possible specific KCO-like effect from Ga.Cr extract. Smooth muscle relaxation via a KCO-like mechanism is known to cause increased K^+^ exit, which leads to membrane hyperpolarization, reduced Ca^2+^ inside the cells, and thus the relaxation of smooth muscles [24, 25]. To confirm this KCO-like effect, Ga.Cr response was obtained in tissues pretreated with a standard potassium channel antagonist/blocker (KCB), glibenclamide, which is partially adenosine triphosphate- (ATP-) dependent KCB [26]. Glibenclamide inhibited the spasmolytic action of Ga.Cr, evident from the rightward shift of the concentration-response curve in rabbit jejunum (Figure 2).

The GI smooth muscle relaxant effect of the extract, possibly mediated via a specific KCO mechanism, was also compared to two standard smooth relaxants, namely, cromakalim and verapamil. Cromakalim is an ATP-sensitive KCO [27] that causes membrane hyperpolarization and muscle relaxation. Verapamil is a CCB that exhibits its smooth muscle relaxant effect via reduced intracellular Ca^2+^ [28]. Being standard spasmolytics, both the agents suppressed the spontaneous contractions of rabbit jejunum (Figure 3). Both cromakalim and verapamil also inhibited the induced contractions from both strengths of K^+^ (25 mM and 80 mM, Figure 3). Of interest is that, similar to Ga.Cr extract, cromakalim, being a KCO, was more specific in blocking 25 mM concentration-induced contractions than the 80 mM contractions. This more potent effect on the K^+^- (25 mM) induced contractions, similar to the extract, was blocked when the tissues were pretreated with glibenclamide, the KCB. Verapamil, on the other hand, inhibited both 25 mM and 80 mM K^+^-induced contractions equipotently. These experiments with the standard drugs clearly differentiated a predominant KCO-like effect from that of a CCB mechanism. This also confirms our finding that Ga.Cr extract mediated its GI smooth muscle relaxant effect via a mixture of KCO and CCB mechanisms with the extract being more specific in the KCO effect. This is why it is more evident in the lower concentration, while the CCB effect is visible in the higher concentrations.

Different studies have been performed on the *Grewia asiatica* fruit to investigate its chemistry. Reports have shown that the fruit contains phenolic compounds such as flavonoids, carbohydrates, proteins and amino acids, tannins, vitamin C, fixed oils, and steroids [3, 15, 29]. From among the class of flavonoid compounds, a number of compounds have been isolated and identified. Some of these are isovitexin, luteolin, nepetin, narirutin, hesperetin, catechin, epigallocatechin, kaempferol, myricetin, quercetin [3, 14, 30], and vitexin [30]. Out of these flavonoids, quercetin, luteolin [31, 32], and catechin [33] have been reported to have a CCB activity. This shows that the CCB activity seen with Ga.Cr can possibly be attributed to these chemicals in the phalsa fruit. As for the KCO effect seen with Ga.Cr, this can most possibly be due to the presence of vitexin in the phalsa fruit. Vitexin is an apigenin flavone glucoside. Similar to Ga.Cr extract, vitexin has been shown to suppress rabbit jejunum contractility in isolated tissues and exhibit glibenclamide-sensitive relaxation of K^+^ (25 mM) contractions while unable to inhibit K^+^ 80 mM contractility [34].

The next round of experiments was conducted on rat aorta to investigate the effect of Ga.Cr on vascular tissues, particularly on vascular resistance. Ga.Cr did not exhibit any constrictor action on the quiescent tone of rat aorta. Once administered on PE-induced contractions, the extract showed concentration-dependent relaxation from 0.3 mg/ml to 10 mg/ml (Figure 4). The vasoconstrictor PE is known to facilitate the entry of Ca^2+^ into the vascular cells through the opening of the receptor-operated Ca^2+^ channel [35]. The rat aorta tissues used in the experiments had intact endothelium. This meant that we could test the involvement of the vasodilator nitric oxide (NO) which is released from the vascular endothelium and is responsible for the regulation of vascular tone [20, 21]. To know if the vasorelaxant action of the extract came about via NO release, the aorta preparations were challenged with L-NAME, a NO synthase blocker [36]. Once the blocker L-NAME was left with the tissues, it ensured that there is no NO synthesis and thus no NO-mediated vascular relaxation. Doing so showed the partial blockade of Ga.Cr extract-elicited vasodilation. This is evident from the rightward shift of concentration-response curves of Ga.Cr extract in Figure 4. This indicated involvement of NO release in the vasodilator action of Ga.Cr.

## 5. Conclusion

The results from this study showed spasmolytic activity of 70% methanol extract of *Grewia asiatica*. This effect was elucidated in isolated small intestinal GI smooth muscle tissues. Ga.Cr medicated this effect through a mixture of KCO and CCB mechanisms, although the extract was more specific for the former than the latter mechanism of action. Ga.Cr also showed vasodilator activity in endothelium-intact rat aorta tissue preparations. Ga.Cr exhibited this action possibly via NO released from vascular endothelium. These results justify the use of *Grewia asiatica* fruit in disorders of the GI and cardiovascular systems. Further studies are needed to run in vivo animal tests and to isolate the pure chemicals from this plant that are responsible for the abovesaid actions.

## Data Availability

The data used to support the findings of this study are included within this article.
